# Age and growth of the endemic groovebelly stingray (*Dasyatis hypostigma*), a heavily exploited batoid in the Brazilian Meridional Margin (21–34° S)

**DOI:** 10.1111/jfb.70305

**Published:** 2025-11-27

**Authors:** Giovanni Arlan Torres, Rodrigo Silvestre Martins, Luciano Gomes Fischer, Luís Gustavo Cardoso, Rodrigo Sant'Ana, Bruno Mourato

**Affiliations:** ^1^ Laboratório de Ciências da Pesca (LabPESCA), Instituto do Mar Universidade Federal de São Paulo (UNIFESP) Santos Brazil; ^2^ Laboratório Integrado de Biologia Marinha (LIBMAR), Instituto de Biodiversidade e Sustentabilidade (NUPEM) Universidade Federal do Rio de Janeiro (UFRJ) Macaé Brazil; ^3^ Laboratório de Dinâmica de Populações Pesqueiras, Instituto de Oceanografia Universidade Federal do Rio Grande (FURG) Rio Grande Brazil; ^4^ Laboratório de Estudos Marinhos Aplicados (LEMA), Escola Politécnica Universidade do Vale do Itajaí (UNIVALI) Itajaí Brazil

**Keywords:** Dasyatidae, elasmobranch, life history, logistic growth model, southwestern Atlantic Ocean

## Abstract

Life‐history data are often lacking for exploited elasmobranchs, jeopardizing proper management and conservation measures for this vulnerable group. Herein, we provided age and growth estimates for the groovebelly stingray (*Dasyatis hypostigma*), a medium‐sized, heavily exploited batoid endemic to the southwestern Atlantic Ocean. Ages were estimated from vertebral centra, including 201 males and 71 females, with sizes varying between 150 and 580 mm in disc width (DW). Observed ages ranged from 0 to 10 years in males and from 0 to 11 years in females. Edge type analysis indicates that band‐pairs are seasonally formed once a year. Among multiple growth models fitted with a Bayesian framework, the logistic model best describes the growth in terms of biological realism and statistical robustness. Females attained larger asymptotic sizes and had lower growth coefficients than males. Growth parameters were comparable to that of *D. hypostigma* from Uruguayan and Argentinian waters, although males seem to grow faster in Brazilian waters.

## 1 INTRODUCTION

The coastal and shelf waters off southeastern and southern Brazil (21–34° S), the so‐called Brazilian Meridional Margin (BMM; Port et al., 2016) comprise a heavily fished demersal environment in the southwestern Atlantic Ocean (Costa et al., 2024; Haimovici, 1998; Port et al., 2016). This vast area is exploited by both artisanal and industrial fleets that use a variety of fishing gears, mostly gillnets and bottom trawls (Port et al., 2016; Vasconcellos et al., 2011). The number of species caught is high (Freire et al., 2021), but, for some groups, difficulties in taxonomic identification and market mislabelling create a problem for appropriate fisheries statistics. Among these, skates and rays have been historically neglected, as they are normally pooled under generic categories dubbed ‘emplastro’ or ‘raias agrupadas’, depending on the region. These labels aggregate several species that are ultimately accounted as single units in the official records (Bornatowski et al., 2018; Schroeder et al., 2024).

The groovebelly stingray (*Dasyatis hypostigma* Santos & Carvalho, 2004) is endemic to the southwestern Atlantic Ocean, ranging from Espírito Santo, Brazil (18° S), down to southern Buenos Aires Province, Argentina (38° S). This stingray inhabits soft bottoms in estuaries and the inner continental shelf at depths up to 80 m, mostly between 5 and 40 m (Santos & Carvalho, 2004). The species is sexually dimorphic, with females larger than males. Maximum recorded size and estimated age are 650 mm in disc width (DW) and 13 years, respectively (Cousseau et al., 2007; Ruocco, 2012). The species is viviparous, and females give birth to two pups per litter. Embryonic development follows the matrotrophic viviparity with lipidic histotrophy (Wourms, 1981). Prey items include small demersal fish and benthic invertebrates (Lemos et al., 2024; Ruocco, 2012). Molecular data indicate a taxonomic homogeneity for the species between 23 and 32° S (Schmidt et al., 2015).

Although not considered a target species, *D. hypostigma* is under intense and unmanaged fishing pressure by both artisanal and industrial fisheries across its distribution range, as this stingray is highly valued due to the quality of its meat (Pollom et al., 2020). The latest International Union for Conservation of Nature (IUCN) assessment classifies the groovebelly stingray as endangered (EN), whereas the most recent Brazilian government assessment places it as data deficient (DD) (ICMBio, 2024; Pollom et al., 2020). There are currently no conservation/management measures towards *D. hypostigma* in Brazil.

Despite the poor records in official fisheries statistics, both molecular and morphological techniques indicate that *D. hypostigma* is the most caught *Dasyatis* species off BMM and probably the most abundant species of this genus in the region (Schmidt et al., 2015). In addition, a recently published catch reconstruction study (Schroeder et al., 2024) estimated that 2503 t of *D. hypostigma* were caught in the region between 2001 and 2009, averaging 250.3 t per year.

In view of the unregulated exploration of the species, accurate estimates of age and growth are required for management purposes. Herein, we examined age and growth of groovebelly stingray collected on the BMM and fitted growth models that could be used in management applications.

## 2 METHODS

### 2.1 Ethics statement

Field sampling was authorized by the Brazilian Institute for Biodiversity Conservation (ICMBio; SISBio clearance numbers 44025‐3 and 68894‐1). Laboratory procedures were approved by UNIFESP's Ethic Committee on Animal Use (CEUA/UNIFESP, clearance number 2772110724).

### 2.2 Sampling and study area

**FIGURE 1:**
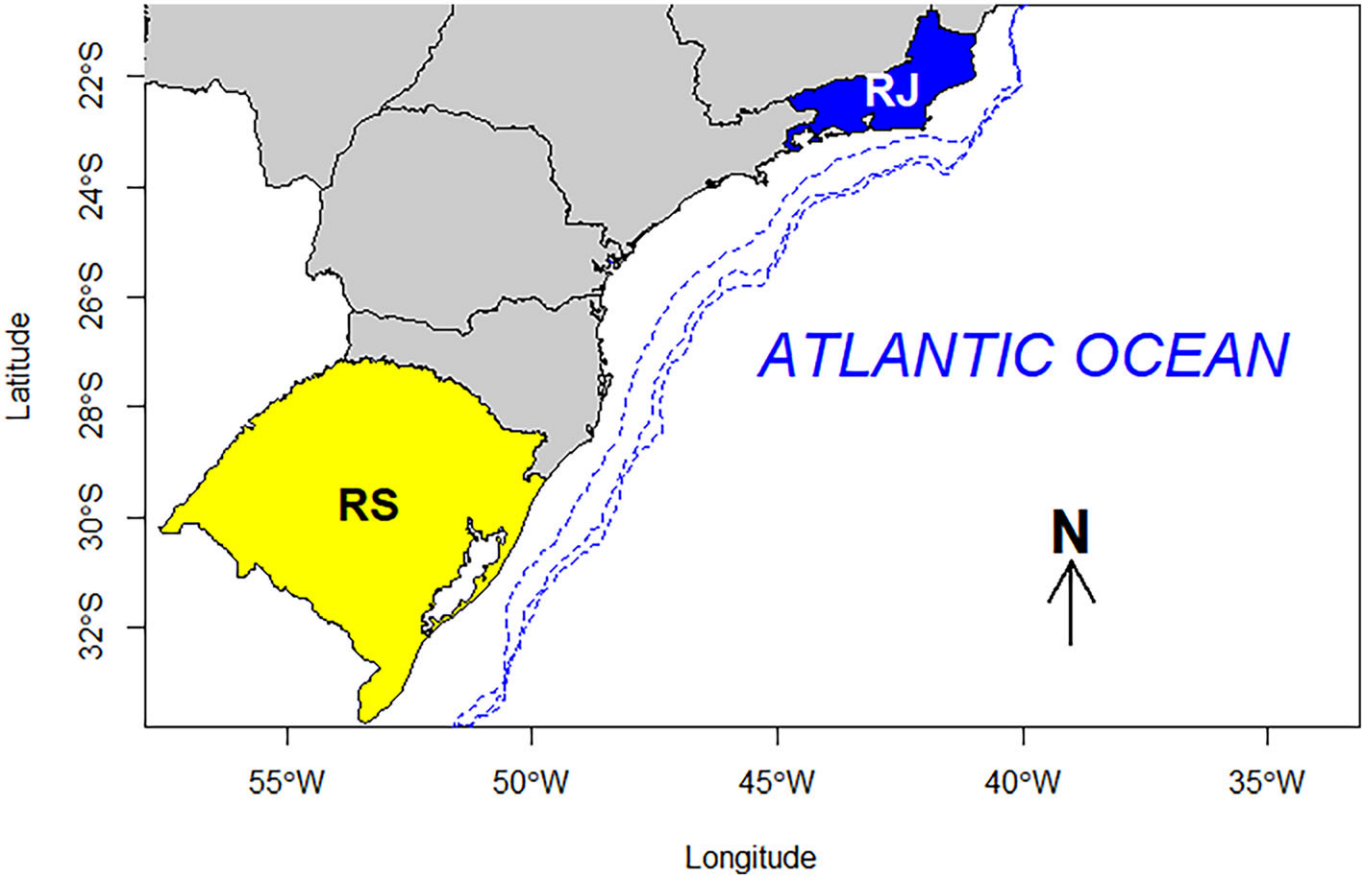
Study area. Brazilian Meridional Margin (BMM; 21–34° S), showing the 100, 150 and 200 m isobaths (dashed lines) and the coasts of Rio de Janeiro (RJ, blue) and Rio Grande do Sul (RS, yellow).

Samples were obtained from industrial pair‐trawl and bottom gillnetting fisheries off Rio de Janeiro (21–23° S) and Rio Grande do Sul (29–34° S) states between May 2022 and April 2024 (Figure 1). Sampling was conducted either onboard during fishing trips or from landings at dockside. In both cases, stingrays were measured ventrally for straight DW (mm), sexed, and a section of 3–10 thoracic vertebral centra were taken from the anteriormost vertebrae of the postcranial skeleton (Cailliet & Goldman, 2004). Vertebral sections were stored in plastic bags identified with a tag with the specimen data (geographic information, calendar day of capture, fishing gear, DW and sex), frozen at −18°C and delivered to the laboratory elsewhere. A summary of examined samples is given in Table 1.

**TABLE 1:** Summary of examined samples of *Dasyatis hypostigma* caught off Brazilian Meridional Margin (21–34° S).

Month and year	Male		Female	
	*n*	DW (mm)	*n*	DW (mm)
May 2022	10	300–440	2	350–520
June 2022	16	180–410	13	210–570
July 2022	7	320–400	4	360–420
August 2022	19	310–420	2	400–410
September 2022	4	300–420	1	380–380
October 2022	8	350–410	6	270–440
November 2022	12	360–465	7	330–510
December 2022	17	270–400	2	350–520
January 2023	11	305–430	3	350–540
February 2023	20	250–400	7	210–485
March 2023	31	250–425	4	305–505
April 2023	27	260–440	17	240–550
May 2023	13	150–400	8	150–580
June 2023	7	310–430	0	‐
April 2024	1	350	1	340
Total	203	150–465	77	150–580

Abbreviation: DW, disc width.

### 2.3 Age assessment

In the laboratory, vertebral sections were thawed at room temperature, thoroughly cleaned with commercial bleach to remove adhered flesh, rinsed with tap water, dabbed on tissue paper and air‐dried. Individual centra were embedded in a polyester resin cutting block and sectioned sagittally through the focus of the centrum using a Buehler Isomet low‐speed rotary saw. Bowtie sections 0.25–0.35 mm thick were obtained using two 102‐mm diamond wafering blades separated by a 0.4‐mm spacer. Each bowtie section was mounted on a black microscopy glass slide using mounting medium (Entellan, Merck). We then observed and photographed the bowtie sections under reflected light using a stereomicroscope at 10–20× magnifications attached to a digital imagery system. Digitalized images were analysed using the ImageJ software (Schneider et al., 2012).

Band‐pairs consisted one wide opaque band and one narrow translucent band (Cailliet et al., 2006). Age was estimated by counting the number of translucent bands, excluding the birthmark (the latter identified as the first distinct band after the focus associated with the change in angle in the intermedialia and corpus calcareum). To avoid subjective reading bias, each sample was identified with a code so that the reader knew neither the size and sex nor the previous age count of each individual (Campana, 2001). Two readings of bowtie sections were made by the same reader in different months to ensure independence of the readings. Age readings were compared and, whenever counts differed, the sample was re‐read. If the difference persisted in the third reading, the sample was excluded from the age analysis.

### 2.4 Relationship between vertebral growth and somatic growth

The usefulness of any hard structure for age and growth studies requires evidence of proportionality between the structure's growth and the somatic growth (Ashworth et al., 2017). We examined the proportionality between the vertebral centrum growth and the somatic growth by fitting a linear regression between the DW and the vertebral radius (VR, the distance from the focus to the outer edge). Differences between sexes were tested using analysis of covariance (ANCOVA) (Zar, 2018).

### 2.5 Age reading precision

Precision of ageing between consecutive readings was calculated using average percentage error (APE; Beamish & Fournier, 1981) and the coefficient of variation (CV; Chang, 1982). Low values for APE and CV indicate high ageing precision. Precision was also visually evaluated using a modified age‐bias plot (Ogle et al., 2023), which considers the mean difference between nonreference and reference ages and performs a one‐sample Student *t*‐test to determine whether mean nonreference age (i.e., *y*‐axis) differed significantly from the reference age for each reference age (i.e., *x*‐axis). In our case, nonreference and reference ages were those obtained in the first and second readings, respectively.

### 2.6 Periodicity of band‐pair formation

The most peripheral band type on each vertebral centrum was classified as either opaque or translucent to determine the frequency of edge types throughout the year. Individuals with only birthmarks were excluded from the analysis because these differ from normal growth band‐pairs. We examined the periodicity of band‐pair formation using the statistical model of Okamura and Semba (2009). In short, this method uses the monthly frequency of opaque bands to identify seasonal cycles of band‐pair formation. This modelling approach tests three hypotheses: (1) no seasonal cycle, with no band formation throughout the months (using a uniform distribution), (2) an annual cycle (when one pair of bands is formed per year, using the von Mises distribution) and (3) a biennial cycle (when two band pairs are formed per season, using a mixture of two finite von Mises distributions). We then used the Akaike Information Criterion (AIC) to determine which model was the best.

### 2.7 Back‐calculation

We back‐calculated size‐at‐age for each sex using the body‐proportional hypothesis (BPH; Francis, 1990), using the following formula:

$${\mathrm{DW}}_{t}=\mathrm{DW}\frac{a+b{\mathrm{VR}}_{t}}{a+b\mathrm{VR}}$$

where DW_*t*_ and VR_*t*_ are the disc width and the vertebra centrum radius at age *t*, and *a* and *b* are the intercept and the slope of the linear regression between DW and VR.

### 2.8 Growth modelling

We paired the estimated age with the respective back‐calculated DWs to fit four distinct growth models, as different growth functions may better describe growth parameters for some elasmobranch species (Dale & Holland, 2012; Gianeti et al., 2019; O'Shea et al., 2013). The three‐parameter von Bertalanffy growth model (VBGM) (Beverton & Holt, 1957) was calculated using the following equation:

$${\mathrm{DW}}_{t}={\mathrm{DW}}_{\infty }\left[1-\exp^{-k\left(t-t_{0}\right)}\right]$$

where DW_*t*_ is the average size at age *t*, DW_∞_ is the asymptotic size, *k* is the annual growth coefficient and *t*_0_ is the theoretical age at size zero.

A two‐parameter VBGM (von Bertalanffy, 1938) was fitted using the size‐at‐birth (DW_0_) intercept rather than the theoretical age at zero, with the equation:

$${\mathrm{DW}}_{t}={\mathrm{DW}}_{\infty }-\left({\mathrm{DW}}_{\infty }-{\mathrm{DW}}_{0}\right)\;\exp^{-\mathrm{kt}}$$

where DW_0_ is the observed size‐at‐birth, and the remaining parameters are as previously defined.

A Gompertz model (Ricker, 1975) was fitted using the following equation:

$${\mathrm{DW}}_{t}={\mathrm{DW}}_{\infty }\;\exp^{-\exp\left(-k\left(t-t_{0}\right)\right)}$$

Finally, we fitted a logistic growth model (Ricker, 1975) with the equation:

$${\mathrm{DW}}_{t}=\frac{{\mathrm{DW}}_{\infty }}{\left(1+\exp^{-k\left(t-t_{0}\right)}\right)}$$

For the Gompertz and logistic models, *t*_0_ is the inflection point of the curve, and the remaining parameters are as previously defined.

Model parameters were estimated using a Bayesian framework in R (R Core Team, 2024). Models were fitted using the brms package (Doll, 2024) with the STAN methodology (STAN Development Team, 2024).

Regardless of the model, a random error term ɛ for individual *i* with a mean of 0 and standard deviation 𝜎 was added:

$${ɛ}_{i}\sim \text{normal}\;(0,\text{𝜎)}$$

We used the following non‐informative prior probability distributions for each parameter:

$$\mathrm{DW}\infty \sim \text{normal}\;\left(0,700\right)$$

$$k\sim \text{normal}\;\left(0,1\right)$$

$$t_{0}\sim \text{normal}\;\left(0,8\right)$$

$$\text{𝜎}\sim {\text{Student}}^{'}s\;t\;\left(3,0,40\right)$$

Because the parameters DW∞ and *k* cannot be negative, they were truncated at zero to yield only positive values. STAN parameterizes the normal distribution with the mean and standard deviation and the Student's *t* distribution with the degrees of freedom, mean and standard deviation.

Each model was implemented by sex using the No‐U‐Turn Sampler (NUTS) with 1000 warm‐up steps to discard and 3000 samples per chain (*n* = 4), totalling 12,000 samples per model. Convergence was assessed visually by inspecting the mixing of fitted parameter chains and performing a posterior predictive check of the fitted model. The R‐hat statistic was also evaluated for model convergence (*R̂* <1.10; Gelman et al., 2013).

The best model was selected based on the lowest AIC. The predicted size‐at‐birth (DW_0_) for each model was also estimated as an additional measure of biological realism (Cailliet et al., 2006; Cailliet & Goldman, 2004). We tested for sex‐specific differences in growth parameters for each model by calculating the difference between posteriors (i.e., *θ*_females_ − *θ*_males_) for each parameter; if the 90% credible interval (CI) did not contain zero, the parameters were considered significantly different (Kéry, 2010).

### 2.9 Longevity

We compared the maximum male and female observed ages with theoretical longevities for each sex following Ricker (1979; 95% DW_∞_) using the formula: 5(ln2)/*k*. We obtained the *k* parameter from the logistic growth model.

## 3 RESULTS

### 3.1 Size and age structure

**FIGURE 2:**
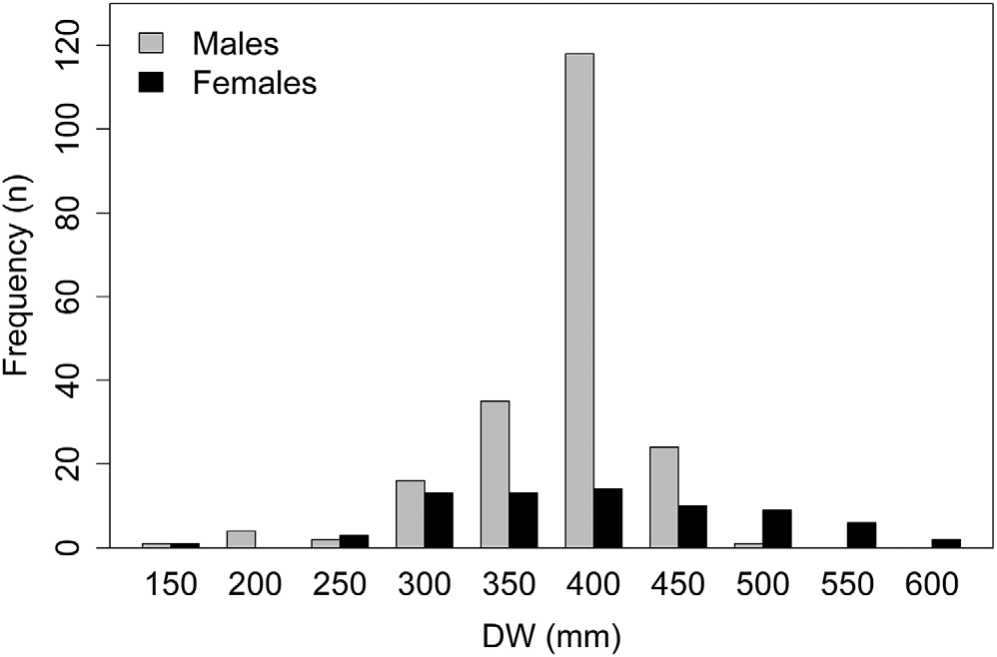
Disc width frequency distribution of *Dasyatis hypostigma* by sex.

From the 280 *D. hypostigma* sampled, we estimated ages for 201 males (162 from Rio de Janeiro and 39 from Rio Grande do Sul) and 71 females (47 from Rio de Janeiro and 24 from Rio Grande do Sul), with sizes varying between 150 and 580 mm DW, totalling 272 individuals (Table 1; Figure 2). Only two males and six females had unreadable vertebrae and were excluded from the analysis. Age‐0 individuals averaged 174 ± 50.3 mm DW (males) and 190 ± 91.4 mm DW (females). The smallest and largest males and females were 150 mm DW (age‐0 individuals) and 465 mm DW (10 years, the largest and oldest male, caught off Rio de Janeiro) and 150 mm DW (age‐0 individuals) and 580 mm DW (11 years, the largest and oldest female, also caught off Rio de Janeiro). The most frequent ages were 1–7 years in males and 1–6 years in females. Males older than 8 years, females older than 6 years and age‐0 individuals (both sexes) were scarce (Table 2).

**TABLE 2:** Comparison between average and back‐calculated (BPH method, Francis, 1990) DW values (mm) of *Dasyatis hypostigma* by age and sex.

Male					Female				
Age	*n*	DW_avg_	*m*	DW_back_	Age	*n*	DW_avg_	*m*	DW_back_
0	5	174	201	213	0	3	190	71	215
1	10	277	196	265	1	10	292	68	272
2	18	311	186	298	2	10	306	58	306
3	24	353	168	328	3	5	349	48	338
4	39	370	144	349	4	11	376	43	363
5	43	383	105	378	5	10	402	32	388
6	30	391	62	365	6	7	416	22	407
7	24	395	32	390	7	0	‐	15	429
8	4	411	8	408	8	2	535	15	457
9	3	418	4	424	9	7	487	13	471
10	1	465	1	456	10	1	505	6	487
					11	5	524	5	511

Abbreviations: DW, disc width; DW_avg_, observed average DW by age on the *n* individuals sampled; DW_back_, back‐calculated average DW by age on the *m* bowtie growth mark readings.

### 3.2 Back‐calculation performance

**FIGURE 3:**
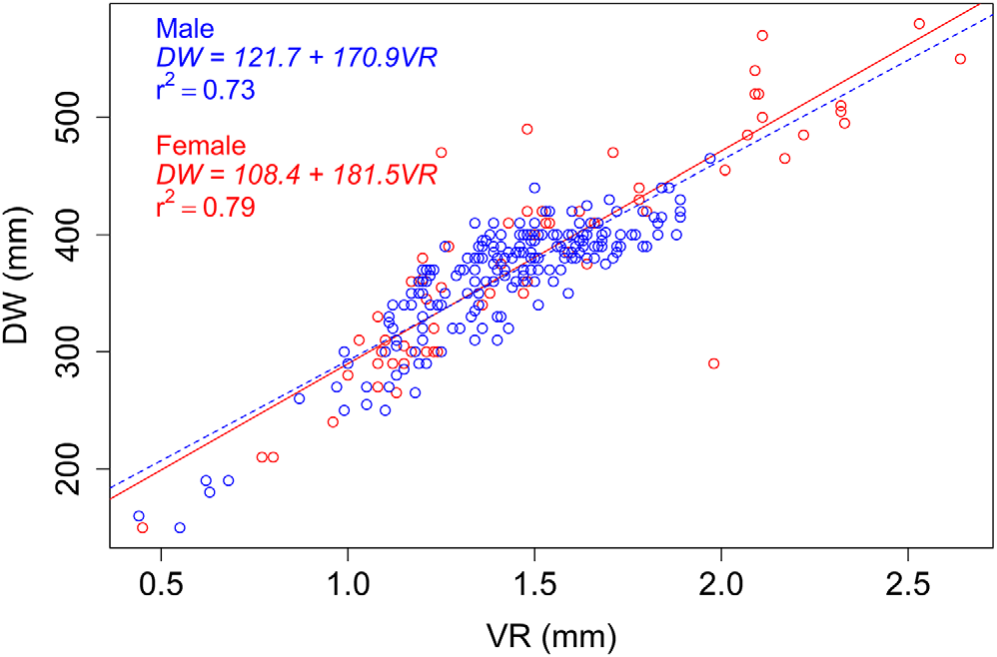
Relationship between disc width (DW) and vertebral radius (VR) for male (blue) and female (red) *Dasyatis hypostigma*.

The relationship between the DW and VR showed no significant differences by sex (ANCOVA, *p* > 0.05; Figure 3), and therefore, a single regression model was used for back‐calculation and posterior growth model fitting. Notwithstanding, a comparison between observed and back‐calculated DW is provided in Table 2. Overall, average back‐calculated DWs were more underestimated in females (15.2 ± 24.3 mm) than in males (6.7 ± 18.0 mm). Interestingly, back‐calculated average sizes for age‐0 individuals were overestimated in both sexes (39 mm in males and 27 mm in females).

### 3.3 Age determination

**FIGURE 4:**
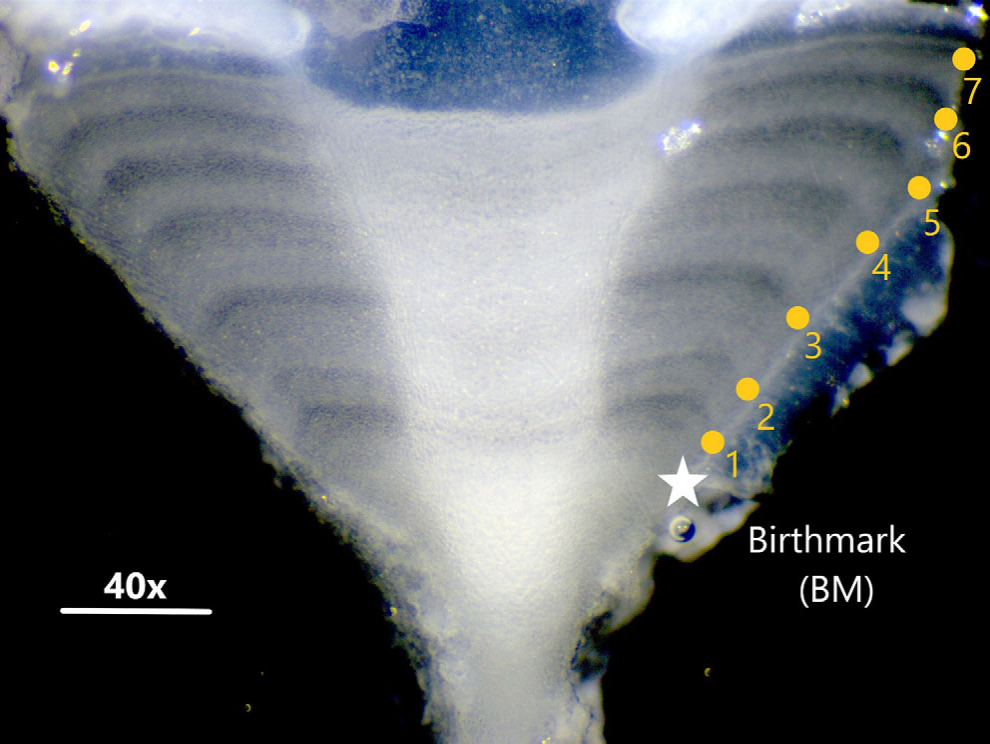
Annual band‐pairs (*n* = 7) formed on the sectioned vertebral centra of a 410 mm DW male *Dasyatis hypostigma*. The yellow circles indicate translucent bands; the white star indicates the birthmark.

Vertebral band‐pairs were clearly visible along the intermedialia and in the corpus calcareum of the vertebral centrum. For practical purposes, band‐pair counts were made on the corpus calcareum (Figure 4). The birthmark was identified as the first distinguishable band after the focus, with a characteristically slight change in the angle of the corpus calcareum. Readable marks ranged from 0 to 10 in males and from 0 to 11 in females. The distance between the birthmark and the vertebral focus was 0.56 ± 0.05 mm in males and 0.58 ± 0.06 mm in females.

### 3.4 Age‐reading precision

**FIGURE 5:**
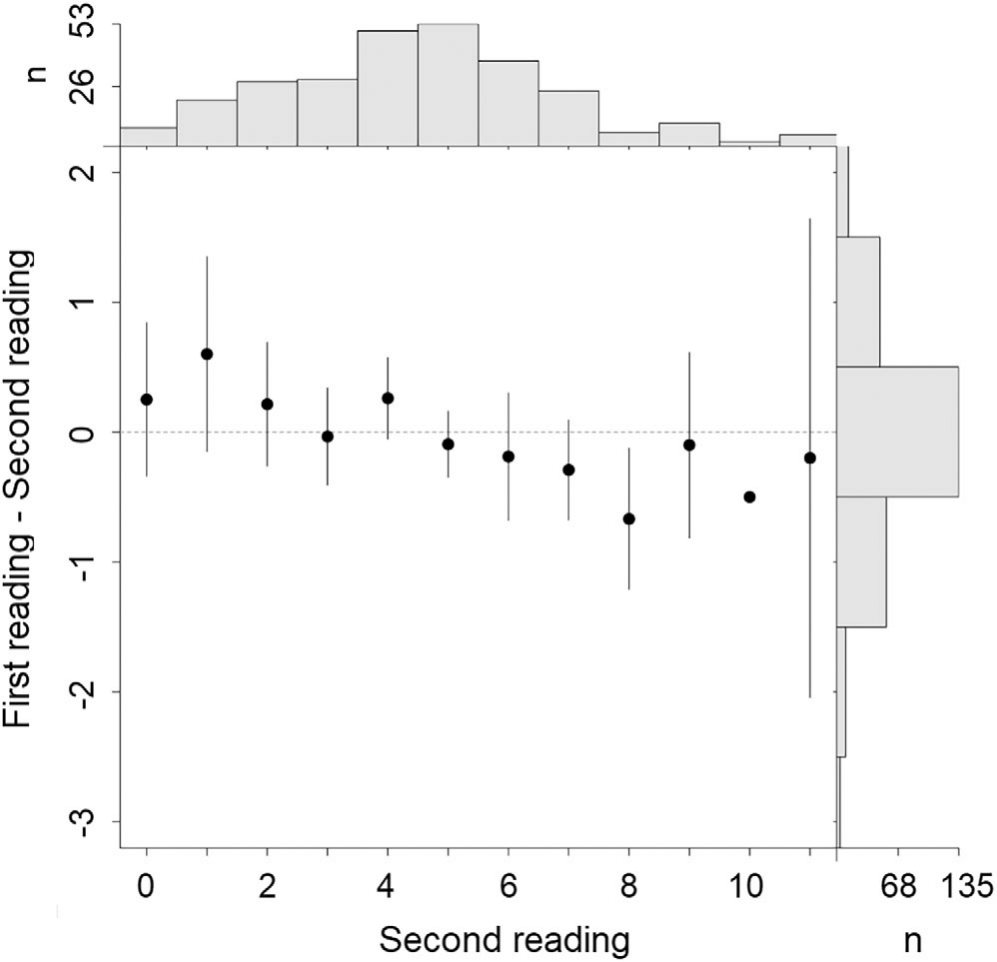
Age‐bias plot for 272 *Dasyatis hypostigma* with age‐specific agreement between two consecutive readings. Vertical bars represent 95% confidence intervals. Marginal histograms above and to the left of the graph represent the sample sizes and the distribution of differences in age estimates. The dashed line at a difference of zero represent the amount of perfect agreement between the sets of age estimates.

First and second reads agreed ±1 year in 87.5% of the bowtie sections examined, resulting in a 9.22% APE and 13.04% CV, respectively. Reading discrepancies higher than 2 years occurred in the remaining 12.5% of the cases. As expected, visual inspection of the age‐bias plot showed consistency between the two readings performed (Figure 5). In addition, no statistical difference was found between the first and second readings (Student's *t*‐test, *p* > 0.05).

### 3.5 Periodicity of band‐pair formation

**FIGURE 6:**
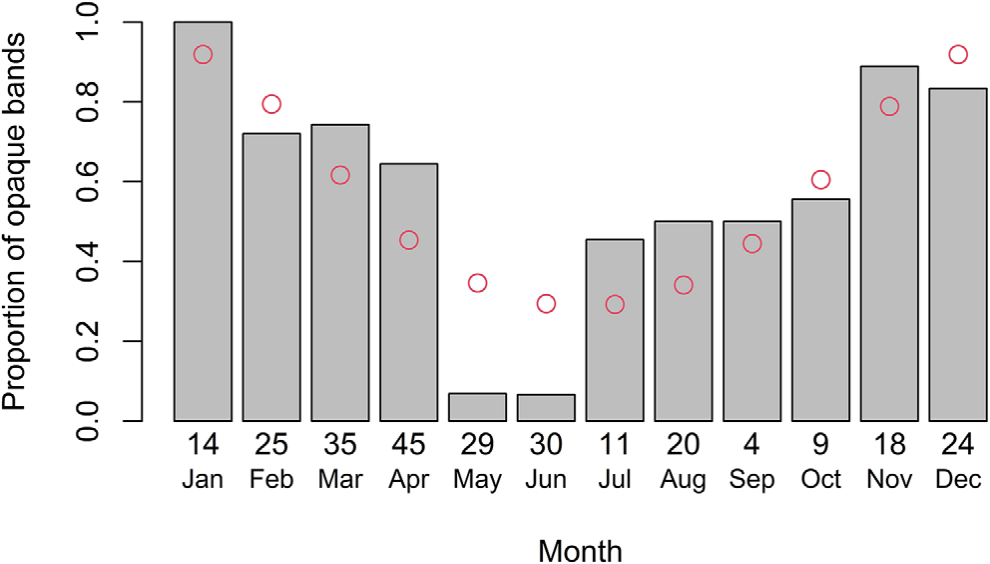
Relative frequency of opaque bands on *Dasyatis hypostigma* vertebral edge by month. Open circles denote values predicted by the centrum edge analysis model. Numbers under bars indicate the sample size for each month.

The monthly frequency of growth bands clearly suggests an annual cycle, with opaque bands deposited during austral spring–summer months, with translucent bands dominating the rest of the year (Figure 6). This is further supported by the lowest AIC value found in Okamura and Semba's one‐peak model (Table 3).

**TABLE 3:** Periodicity of band‐pair deposition for *Dasyatis hypostigma* off BMM using the no seasonal cycle (zero peak), annual cycle (one peak) and biannual cycle (two peak) models according to AIC values.

Model	AIC	Δ_*i*_	*w*_*i*_ (%)
One peak	299.63	0.00	100
Two peak	353.16	53.56	0
Zero peak	363.59	63.99	0

Abbreviations: Δ_*i*_, Akaike differences; AIC, Akaike Information Criterion; *w*_*i*_, Akaike weights; BMM, Brazilian Meridional Margin.

### 3.6 Growth modelling

**FIGURE 7:**
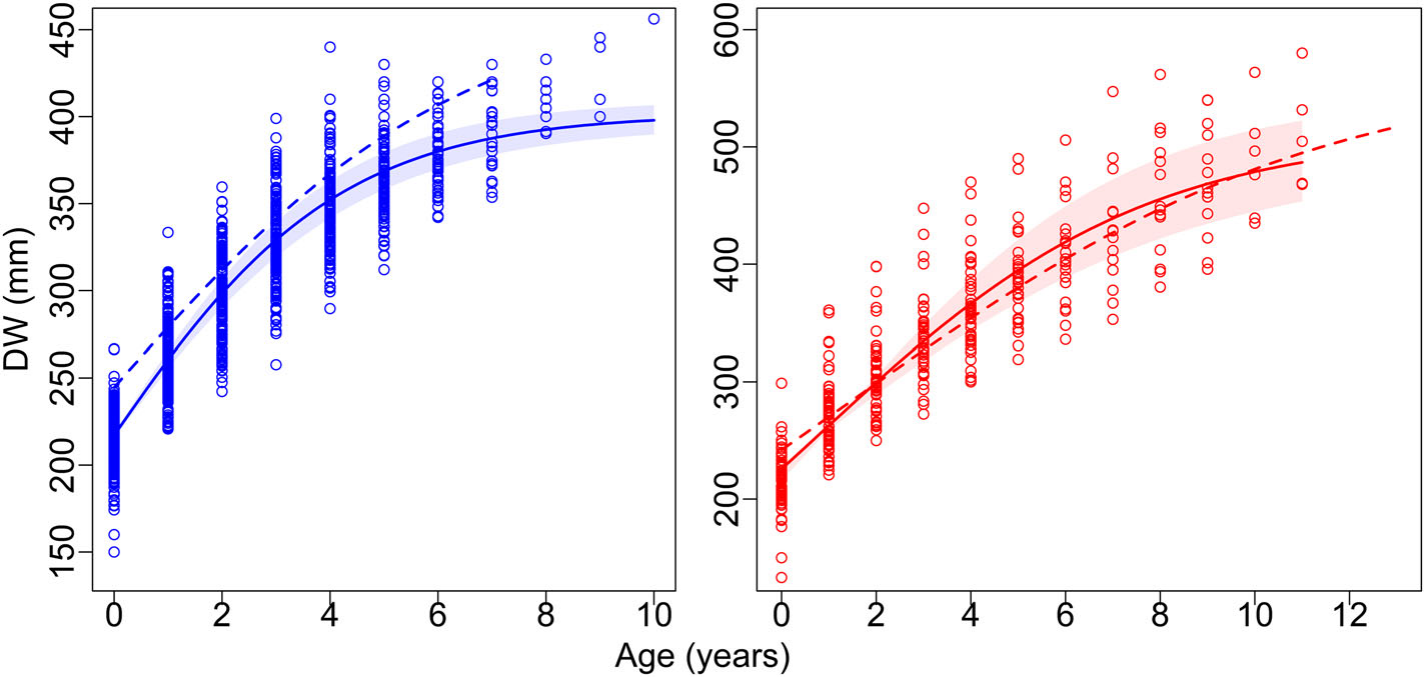
Growth function fits for male (left) and female (right) *Dasyatis hypostigma* off Brazilian Meridional Margin (BMM) (continuous line) and off Uruguayan‐Argentinian coast (dashed line). The shaded area indicates the credibility interval (*α* = 0.05).

Model parameters of the four growth models fitted to the data are given in Table 4, and fitted curves for the best models are illustrated in Figure 7. All models fitted displayed good convergence (*R̂* = 1.0). The logistic growth model yielded the best statistical fit for both males and females, with the lowest AIC values. Curve fitting was better in males, which had a narrow credibility interval across the whole range of sizes available. The two‐ and three‐parameter VBGM were the second best for males and females, respectively.

**TABLE 4:** Growth parameter estimation and goodness‐of‐fit measure for *Dasyatis hypostigma* off southeastern–southern Brazil.

	DW_0_ (mm)	DW_∞_ (mm)	*k* (year^−1^)	*t*_0_	AIC
Male					
VBGM2		388.95 ± 3.46 (382.50–395.88)	0.45 ± 0.02		0.031
VBGM3	212.53	426.76 ± 6.20 (415.32–439.52)	0.25 ± 0.01	−2.77 ± 0.12	0.157
Gompertz	216.12	411.26 ± 4.68 (402.57–420.88)	0.35 ± 0.02	−1.26 ± 0.04	0.087
Logistic	217.08	401.70 ± 3.85 (394.67–409.41)	0.45 ± 0.02	−0.36 ± 0.04	−0.042
Female					
VBGM2		506.15 ± 13.4 (481.51–534.08)	0.22 ± 0.02		−0.001
VBGM3	228.18	588.96 ± 34.0 (532.78–663.92)	0.13 ± 0.02	−3.77 ± 0.34	−0.109
Gompertz	224.04	537.03 ± 19.8 (503.86–581.29)	0.21 ± 0.02	−0.64 ± 0.14	0.025
Logistic	225.80	512.22 ± 14.6 (486.48–543.97)	0.29 ± 0.02	0.82 ± 0.21	−0.138

*Note:* Values of DW∞ are means ±1 standard deviation and the 95% credible interval (in parenthesis).

Abbreviations: AIC, Akaike's Information Criterion; DW_0_, predicted size at birth; DW∞, asymptotic size; *k*, growth coefficient; *t*_0_, theoretical age at size 0; VBGM2, two‐parameter von Bertalanffy growth model; VBGM3, three‐parameter von Bertalanffy growth model.

**FIGURE 8:**
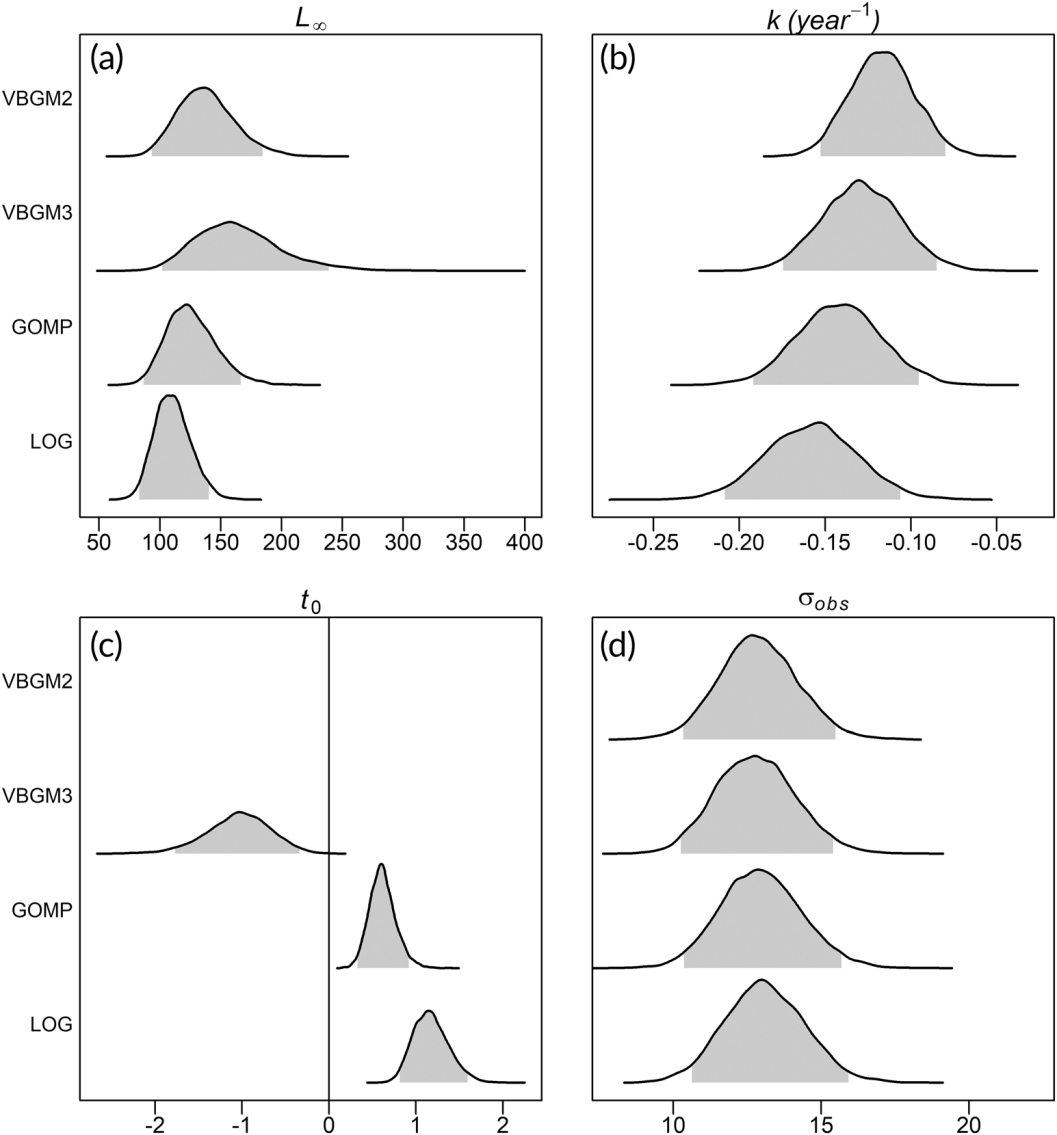
Posteriors of the parameter differences by sex in *Dasyatis hypostigma*. (a) asymptotic length, DW∞; (b) body growth coefficient, *k*; (c) theoretical age at size zero, *t*_0_; (d) observation error, σ_obs_. VBGM2, two‐parameter von Bertalanffy growth model; VBGM3, three‐parameter von Bertalanffy growth model; GOMP, Gompertz growth model; LOG, logistic growth model. The shaded area indicates the credibility interval (*α* = 0.05).

Modelled asymptotic sizes in the best models (males: 402 mm DW and females: 512 mm DW) were smaller than the largest stingrays sampled (males: 465 mm DW, females: 580 mm DW). In contrast, estimates of size‐at‐birth (DW_0_) were always larger than those observed in our samples. Notwithstanding, regardless of the model, predicted DW_0_ values were closer to mean back‐calculated sizes‐at‐birth (213 mm DW for males and 215 mm DW for females). Because the 90% CI of the posterior difference between female and male did not contain zero for all parameters (Figure 8), one can conclude that there were statistically significant sex‐specific differences for all parameters for all models fitted (*p* < 0.05).

### 3.7 Growth comparisons among species of the family Dasyatidae

**FIGURE 9:**
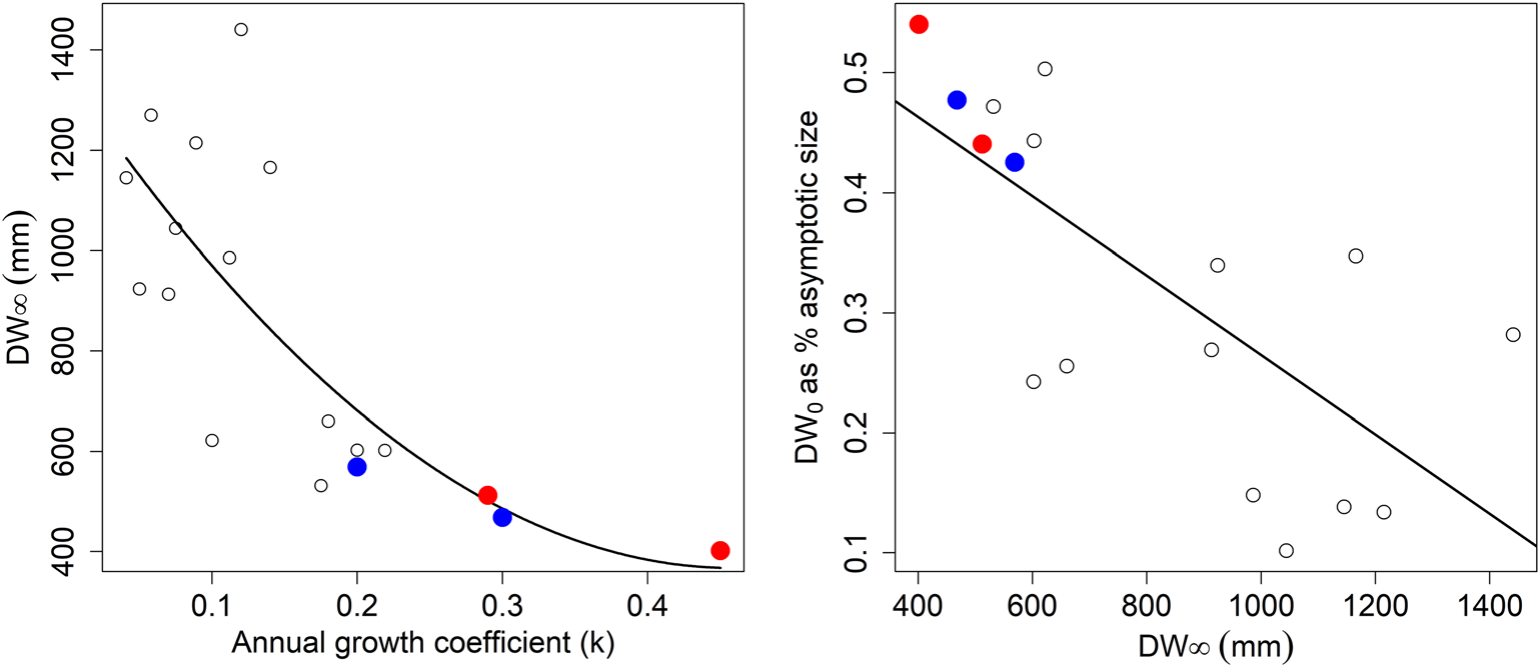
Relationships between the asymptotic size and the growth coefficient (left) and between the size‐at‐birth as percentage of the asymptotic size against the DW∞ (right) in dasyatids. The unrealistic *k* and DW_0_ estimations for Agean Sea *Dasyatis pastinaca* and *Dasyatis marmorata* were not included in these plots. A polynomial curve with the formula DW∞ = 1352.7–4289.2 *k* + 4666.2 *k* ^2^ (Spearman's ρ = −0.76, *p* < 0.001) best describes the relationship between DW∞ and *k*, whereas a negative linear trend was found between DW_0_ expressed as a percentage of DW∞ and DW∞ (formula: DW_0_% DW∞ = 5.96 × 10^−1^ – 3.31 × 10^−4^ DW∞, Spearman's ρ = −0.75, *p* < 0.001). Blue and red circles represent Uruguayan‐Argentinian and Brazilian *Dasyatis hypostigma*, respectively.

A comparison between our growth parameters and some selected dasyatids is provided in Table 5 and Figures 7 and 9. Most growth curves stick to the three‐parameter VBGM, followed by the logistic model, with a two‐parameter VBGM appearing just once. Minimum and maximum observed longevity were 5 and 28 years, respectively (Table 5).

**TABLE 5:** Comparison of growth parameters among selected dasyatid stingray species.

Species	Size	Region	Model	Sex	DW_0_	DW∞	*k*	*t*_0_	Age_max_	*n*	Reference
*Dasyatis hypostigma*	Medium‐sized	Brazilian Meridional Margin	Logistic	Male	217.0	401.7	0.45	−0.36	10	201	This study
				Female	225.8	512.2	0.29	0.82	11	71	
		Uruguay‐Argentina	Logistic	Male	223.5	468.2	0.3	0.3	7	22	Ruocco, 2012
				Female	242.1	568.9	0.2	1.5	13	29	
*Dasyatis pastinaca*	Large‐sized	Mediterranean Sea	VBGM3	Pooled	162.6	1215.0	0.089	−1.615	10	256	Ismen, 2003
				Pooled	106.2	1044.3	0.075	−1.43	13	384	Girgin & Başusta, 2016
				Male	158.4	1145.4	0.041	−3.63	13	175	
				Female	106.5	1270.6	0.058	−1.51	13	209	
	Medium‐sized	Aegen Sea	VBGM3	Pooled	7.4	582.8	0.06	−0.213	13	72	Yeldan & Gundogdu, 2018
*Dasyatis marmorata*	Medium‐sized	Aegen Sea		Pooled	31.8	460.9	0.36	−0.162	13	143	Yeldan & Gundogdu, 2018
*Bathytoshia lata*	Large‐sized	Hawai'i	Logistic	Male	405.0	1166.0	0.14	4.52	25	88	Dale & Holland, 2012
				Female	406.0	1441.0	0.12	7.61	28	115	
*Hypanus dipterurus*	Medium‐sized	Pacific Mexico	VBGM3	Male	313.0	622.0	0.1	−6.8	19	148	Smith et al., 2007
	Large‐sized			Female	314.0	924.0	0.05	−7.61	28	191	
*Dasyatis chrysonota*	Medium‐sized	Eastern South Africa	VBGM3	Male	251.1	531.8	0.175	−3.65	5	105	Cowley, 1997
	Large‐sized			Female	245.9	913.4	0.07	−4.48	7	165	
*Hypanus guttatus*	Medium‐sized	Ceará NE Brazil	VBGM2	Male	145.0	602.2	0.219		9	95	Gianeti et al., 2019
	Large‐sized			Female	145.0	986.1	0.112		14	101	
	Medium‐sized	Pernambuco NE Brazil	VBGM3	Pooled	168.7	660.1	0.18	−1.64	13	203	Vieira, 2013
		Maranhão NE Brazil		Pooled	267.3	602.8	0.2	−2.93	15	59	

Abbreviations: Age_max_, maximum observed age; DW_0_, predicted size at birth; DW∞, asymptotic size; *k*, growth coefficient; large‐sized, DW∞ > 700 mm; medium‐sized, DW∞ < 700 mm; *t*_0_, theoretical age at size zero (VBGM) or the inflection point of the curve (Gompertz and logistic models); VBGM2, two‐parameter von Bertalanffy growth model; VBGM3, three‐parameter von Bertalanffy growth model.

A visual comparison of growth curves between Brazilian and Uruguayan‐Argentinian male *D. hypostigma* indicates a faster growth in Brazilian waters, whereas growth trajectories of females were similar, as the Uruguayan‐Argentinian curve overlaps with the credibility interval of the Brazilian stingrays (Figure 7).

The DW∞, *k* and *t*_0_ of stingray species comparable in size to the Brazilian *D. hypostigma*, that is, *D. hypostigma* from Uruguayan‐Argentinian waters, *Dasyatis marmorata* (Steindachner, 1892) and *Dasyatis pastinaca* (Linnaeus, 1758) from the Mediterranean Sea and *Hypanus guttatus* (Bloch and Schneider, 1801) from the Pernambuco and Maranhão coasts, NE Brazil, ranged from 401 to 660 mm, 0.18 to 0.36 year^−1^ and − 2.93 to 0.8 year. Interestingly, growth parameters of some males of sexually dimorphic large‐sized species [i.e., *H. guttatus* from Ceará coast (NE Brazil), *Hypanus dipterurus* (Jordan and Gilbert, 1880) from Pacific Mexico and *Dasyatis chrysonota* (Smith, 1828) from eastern South Africa] were closer to those of medium‐sized dasyatids. On the contrary, the growth coefficient reported for Agean Sea *Dasyatis pastinaca* (0.06 year^−1^) is likely flawed, as low *k* values in dasyatids are associated with large‐sized species (Table 5). As expected, the growth coefficient had a negative relationship with the asymptotic size, with most values ranging between 0.1 and 0.45 for medium‐sized species and between 0.041 and 0.14 for larger species (Figure 9).

Estimated size‐at‐birth ranged from 7.4 to 406 mm DW, with the smallest estimations (for *D. pastinaca* and *D*. *marmorata* from the Aegean Sea; Table 5) being clearly unrealistic. A plot of the DW_0_ as percentage of the asymptotic size against the DW*∞* had a negative linear trend, with the most DW_0_ estimations for medium‐sized species (including southwestern Atlantic *D. hypostigma*) ranging between 42% and 54% of the DW*∞*, whereas values were lower than 35% DW*∞* in large species (Figure 9).

### 3.8 Longevity

Estimations at which males and females reach 95% of the DW∞ were reasonably closer to observed ages in both models, differing only by ~2 years in males (7.7 years estimated versus 10 years observed) and 1 year in females (12 years estimated versus 11 years observed).

## 4 DISCUSSION

### 4.1 Overview

*D. hypostigma* figures among the most landed batoids in the BMM by both artisanal and industrial fisheries, and strong evidence suggests that it may actually be the most important stingray species in the catches (Schmidt et al., 2015; Schroeder et al., 2024). Given the lack of growth studies directed towards *D. hypostigma* in Brazilian waters, timely estimation of growth parameters, such as those presented in this study, is undoubtedly important for a proper management and conservation of this endemic stingray.

### 4.2 Age estimation and periodicity of growth band‐pair formation

Band‐pairs on sectioned vertebrae were clearly visible in *D. hypostigma*, and it seems to conform to the general pattern observed in other dasyatids (e.g., *H. guttatus*; Gianeti et al., 2019), including *D. hypostigma* in the southernmost extreme of its distribution area (Ruocco, 2012).

Age‐reading precision metrics, such as APE and CV, usually fall in values of ~5.5% and <7.6%, respectively (Campana, 2001). We obtained values of 9.2% (APE) and 13.0% (CV), which are close to the values reported by Ruocco (2012) for Uruguayan‐Argentinian *D. hypostigma* (APE: 11%, CV: 16%), and also in line with other studies, such as *H. dipterurus* (APE: 9.8%, CV: 13.1%; Smith et al., 2007), *Maculabatis astra* (Last, Manjaji‐Matsumoto and Pogonoski, 2008) (APE: 7.8%, CV: 11.0%), *Neotrygon picta* (Last and White, 2008) (APE: 7.1%, CV: 10.1%) (Jacobsen & Bennett, 2010), *Neotrygon annotata* (Last, 1987) and *Neotrygon kuhlii*, (Müller and Henle, 1841) (APE: 7.5%, CV: 10.6% for both species; Jacobsen & Bennett, 2011). In addition, our age‐bias plot suggests good consistency between successive readings. Thus, judging from our results, it seems that we achieved reasonable age readings for Brazilian *D. hypostigma*.

We used the cyclic model of Okamura and Semba (2009) to investigate the periodicity of growth band‐pair formation in *D. hypostigma*, as traditional methods, such as the marginal increment analysis (MIA; Cailliet, 1990), yielded no meaningful results (i.e., neither monthly nor seasonal signals; data not shown). This pattern is also verified in other subtropical and tropical dasyatids, such as *H. guttatus* (e.g., Gianeti et al., 2019), although a clear seasonal MIA is reported for *Bathytoshia lata* (Garman, 1880) off Hawai'i (Dale & Holland, 2012). This suggests that the pattern of band‐pair deposition and growth varies based on the species in elasmobranchs (Lessa et al., 2006; Okamura & Semba, 2009).

Band‐pair formation was clearly seasonal, with the lowest and highest proportions of opaque bands observed during autumn–winter and spring–summer, respectively. Band‐pair formation is not always associated with seasonal temperature fluctuations in elasmobranchs and may be related to other factors, such as reproduction and feeding (Dale & Holland, 2012; Natanson, 1993). In fact, parturition in *D. hypostigma* seems to take place in autumn–winter months, as suggested by the appearance of age‐0 individuals in our samples at this time of the year (data not shown), meaning that females likely get pregnant some 4 months earlier, during spring–summer (Hamlett et al., 1993). Additionally, spring–summer subsurface intrusions of nutrient‐rich South Atlantic Central Water into the BMM boost regional benthic biological productivity (Borzone et al., 1999), likely providing an improved feeding ground for *D. hypostigma* and other demersal fishes. Thus, reproduction and feeding opportunities may partially explain the spring–summer faster growth and the band‐pair formation pattern found in the present study.

### 4.3 Growth modelling

Growth models other than the classic (i.e., three‐parameter) VBGM are being increasingly applied in elasmobranchs (Dale & Holland, 2012; Gianeti et al., 2019;O'Shea et al., 2013; Smart & Grammer, 2021). Although the VBGM is often fitted, alternative models such as the two‐parameter VBGM, Gompertz and logistic models may better explain body growth in some species. In the specific case of dasyatid stingrays, growth in tropical species has been shown to be best described by the two‐parameter VBGM (Gianeti et al., 2019; O'Shea et al., 2013), whereas others conform to the logistic model (e.g., *B. lata*; Dale & Holland, 2012). Clearly, whenever possible, a multi‐model approach should be encouraged to find the best species‐specific fits (O'Shea et al., 2013; Smart & Grammer, 2021).

The logistic growth model produced the best fits and most realistic biological parameters for male and female Brazilian *D. hypostigma*, in line with the growth modelling of Uruguayan‐Argentinian *D*. *hypostigma* (Ruocco, 2012). In addition, growth rates were faster in males than in females, with the latter reaching larger asymptotic sizes in both studies, a pattern also described in other dasyatids (Cowley, 1997; Gianeti et al., 2019; Girgin & Başusta, 2016; Smith et al., 2007).

Although *D. hypostigma* female growth was found to be similar between Brazilian and Uruguayan‐Argentinian stingrays, our data suggest that males grow faster in Brazilian waters. However, such comparisons must be taken with reserves because of the small sample sizes and the use of different statistical methods (i.e., Bayesian versus frequentist) between ours and Ruocco's (2012) studies. Yet, a Bayesian approach, such as employed here, should yield improved (or equivalent) results to a frequentist approach and must be encouraged in elasmobranch age and growth studies (see Smart & Grammer, 2021).

Notwithstanding the results obtained for the logistic model, the three‐parameter VBGM is the most used model in fisheries biology, as the parameters are classical inputs for stock assessment models (Gianeti et al., 2019). Therefore, the three‐parameter VBGM may be regarded as the most adequate model for *D. hypostigma* stock assessment if the species becomes the subject of fishing management efforts in the future.

### 4.4 Longevity

Estimated theoretical longevities were comparable to observed ages in *D. hypostigma* of both sexes, suggesting not only that we achieved reasonable age estimations but also that we obtained a representative sample of the oldest stingrays available in the study area. In addition, such a match further supports the logistic growth function as the best model. An agreement between observed ages, theoretical longevities and the logistic growth function as the best model is also reported for *B. lata* off Hawai'i (Dale & Holland, 2012).

Our results partially contrast with the findings of Ruocco (2012) for *D. hypostigma* sampled on the Uruguayan‐Argentinian coast, where the theoretical longevity (also obtained from a Ricker's, 1979 model, as in the present study) overestimated the observed age in males by 7 years. Such discrepancy indicates a sampling bias for males in that study. On the contrary, the theoretical longevity and the observed age reported by the author differed only by 1 year in females.

### 4.5 Limitations

Although the use of back‐calculated data for growth model fitting was justifiable due to the low number of individuals sampled in this study, particularly in the case of females, some critical points must not be overlooked.

Our samples were collected by bottom trawl and gillnets and, judging from the pattern observed in the size frequency given in Figure 2 (most individuals concentrated between 300 and 450 mm DW), and the scatterplot shown in Figure 3, it appears that many individuals were caught by gillnetting. This indicates that our model parameters may have been influenced by the selectivity of this fishing gear. Thus, sampling must be planned to reduce or eliminate the effect of selectivity in future studies.

The back‐calculated female growth curves had a wide DW∞ credibility interval (widest in the three‐parameter VBGM), which suggests that a larger and more representative sample of females is no doubt necessary to improve growth model fitting. We hypothesize that our male‐biased sampling may be related to spatiotemporal segregation of sexes. Actually, although males overnumbered females in all calendar months (see Table 1), a higher proportion of females was found during austral autumn–winter, coinciding with the appearance of age‐0 individuals in our samples, suggesting the use of the fishing grounds for parturition. This pattern of habitat use by sex has been found in other dasyatids [e.g., *Dasyatis brevicaudata* (Hutton, 1875); Le Port et al., 2012].

Modelled size‐at‐birth (DW_0_) was larger than the observed average found in our samples for both sexes. This would violate the ‘biological realism concept’ in elasmobranch age and growth studies recommended in Cailliet and Goldman (2004) and Cailliet et al. (2006). This concept emphasizes that the best growth models should not only be statistically robust but also biologically meaningful. However, the differences may reflect a sampling artefact, as the smallest individuals found may comprise abortions due to capture‐induced parturition (Adams et al., 2018). Although improved sampling and ageing of the smallest free‐swimming individuals available in the wild are certainly necessary to test this hypothesis, we do have age‐0 individuals as large as 210 mm DW in our samples (data not shown), thus closer to our modelled DW_0_ (see Tables 4 and 5). In addition, our estimations were in line with the values reported for medium‐sized dasyatids (see Table 5 and Figure 9).

## 5 CONCLUSIONS

In summary, we provided, for the first time ever, baseline information on the life‐history parameters of *D. hypostigma* on the BMM. Both male and female stingrays had their growth best described by the logistic model. The relatively short longevity (10 and 11 years in males and females, respectively, implying in a fairly fast‐generation time) plus the estimated growth coefficients (which figure among the largest observed in medium‐sized dasyatids) suggest that *D. hypostigma* may be more resilient to fishing pressure than larger and slower‐growing species. However, given the unregulated and uncontrolled exploitation of *D. hypostigma* in Brazilian waters (Pollom et al., 2020), directed exploitation towards this stingray must not be encouraged until a robust stock assessment is achieved and proper management measures are in place.

## AUTHOR CONTRIBUTIONS

Giovanni Arlan Torres contributed with primary data acquisition and analysis and manuscript preparation; Luciano Gomes Fischer contributed with funding, data collection and manuscript review; Luís Gustavo Cardoso contributed with data collection and manuscript review; Rodrigo Sant'Ana contributed with data analysis and manuscript review; Rodrigo Silvestre Martins contributed with ideas, mentoring, data analysis and manuscript preparation; Bruno Mourato contributed with ideas, data analysis and manuscript review.

## FUNDING INFORMATION

The Fundo Brasileiro para a Biodiversidade (FUNBIO) supported field sampling and a FUNBIO grant to Giovanni Arlan Torres through the SALVAR Project – Science for shark conservation off southeastern and southern Brazil.
